# Hyperemesis Gravidarum is associated with substantial economic burden in addition to severe physical and psychological suffering

**DOI:** 10.1186/s13584-016-0099-y

**Published:** 2016-10-10

**Authors:** Jone Trovik, Åse Vikanes

**Affiliations:** 1 Department of Clinical Science, University of Bergen, Bergen, Norway; 2 Department Obstetrics and Gynaecology, Haukeland University Hospital, Jonas Liesvei 72, 5021 Bergen, Norway; 3 Meidell-Vikanes Gynaecological Consultation, Oslo University Hospital, Oslo, Norway; 4 The Intervention Centre, Oslo University Hospital Rikshospitalet, Oslo, Norway

**Keywords:** Antiemetics, Cost, Day-care, Hyperemesis gravidarum, Nausea and vomiting in pregnancy, Nutrition, Quality of life

## Abstract

Hyperemesis gravidarum (HG) affects 1 % of all pregnant women and in western societies it is the most common cause for hospital admission during first trimester. The economic burden of the disease has barely been studied. To estimate the Israeli national burden of HG, Konikoff and co-workers obtained data retrospectively on hospital costs as well as loss of workdays from 184 women hospitalized due to HG from December 2010 until December 2013. Their findings emphasise the need for better treatment to reduce the burden of this disease both for the individual as well as the society.

## Background

The most extreme form of nausea and vomiting in pregnancy (NVP); Hyperemesis Gravidarum (HG), severely affects women’s wellbeing and inhibits performance of normal daily living, including work, whether paid or unpaid as family/house caretakers [1]. Women with HG often require hospitalization, explaining why this condition is the most common reason for hospitalization during the first trimester of pregnancy [2, 3]. The etiology of HG remains unknown, although some risk factors have been identified. HG is inherited from mother to daughters and are twice as common in monozygotic as dizygotic twins, suggesting a genetic component [4–6]. Helicobacter pylori infection is the most prevalent environmental factor [7, 8]. Physicians have also been afraid of treating HG patients, given the fact that Thalidomide was given women suffering from NVP in the 1950s causing limb deformities in thousands of babies [9].

Studies exploring the economic burden of HG are sparse. Thus the study from Konikoff and co-workers [10] is clearly welcomed.

In the Konikoff study, data obtained from a 3-years cohort comprising 184 women hospitalized due to HG at the Galilee Medical Center and the hospital’s birth registry were used to estimate the incidence of HG; 1.2 %. Based on the number of days in hospital (mean 2.2) and post-hospital rest days (mean 4.6), the total annual cost in the Western Galilee was estimated to be approximate 453 thousand NIS (110 thousand USD). When taking into account the nearly 171,000 yearly deliveries in Israel per year, the total national economic burden due to HG was estimated to be 20 million NIS (approximately 5.2 million USD).

This is a large sample study, covering hospitalization due to HG over three years and the first study to elucidate HG in this region. The focus on the economic impact of HG is important.

## Context

The estimated incidence of 1.2 % in this Northern Israeli hospital cohort [10] is well within rates reported from other industrialized countries; Norway 1.1 % [11], USA 1.2 % [12], England 1.5 % [13] and Japan 3.6 % [14].

In Norway between 0.8 to 3.2 % women (or pregnancies) develop HG, where the prevalence is found to differ according to maternal country of birth. Primiparous women born in India and Sub-Saharan Africa were three times more likely to develop HG compared to those born in Norway [15]. These differences could not be explained by differences in socio economic factors, such as maternal length of education. Differences in help-seeking behaviour or differences in availability of health service could, however, partly explain these findings; although in Norway health services are based on equality and are free of charge. Moreover, both genetic and lifestyle factors are believed to contribute to the development of HG, but the etiology remains mostly unknown [16]. While the study from Konikoff [10] did not reveal any significant difference in incidence of HG between women of Arabic and Jewish ethnicity, this can either be seen as weakening the genetic risk hypothesis or suggest that the women of Arabic and Jewish origin studied are genetically closely related. Alternatively, since HG is inherited from mothers to daughters, the results may reflect common environmental factors transferred along the maternal line; i.e. lifestyle factors such as diet, helicobacter pylori etc. An earlier study from southern Israel [17] estimated an overall incidence of HG as 0.6 %, but in relation to the total birthing population Jewish women had significantly higher risk of hospitalization compared to the Arabic Bedouin population. We interpret this to most likely represent cultural or environmental differences rather than disease incidence.

The Konikoff-study has some limitations: The Israeli Jewish population comprise several different sub groups due to immigration from Eastern Europe, including USSR, and North Africa; this complicates interpretation of ethnic differences - which was a main outcome in their study. Information on education, known to reflect socio economic status, is valuable when exploring differences between ethnic groups. Not having this information is considered a limitation; also because HG is associated with length of education. Moreover; HG is also associated with maternal smoking habits, which have been suggested to differ between the two ethnic groups. Not having accurate information of smoking habits may have influenced the study’s findings.

HG is known to reduce women’s quality of life (QOL) in the short term [18]. One study compared the intensity of symptoms among 160 pregnant women at 11 weeks to the symptoms of nausea and vomiting experienced by patients receiving chemotherapy [19]. The findings showed that the intensity of “normal” nausea and vomiting at 11 weeks was comparable to the kind of nausea patients experience in the wake of moderately nausea-producing chemotherapy. Whether HG has consequences for women’s long-term quality of life has not yet been studied. Previous studies have, however, showed that women with severe HG are less able to welcome another pregnancy and that they are known to consider terminating their next pregnancy due to previous suffering [20, 21].

The specific pregnancy unique questionnaire of emesis (PUQE) has been validated in several settings and languages [22, 23]. Three questions quantify nausea, retching and vomiting and sum up as a PUQE-score from 3 (no NVP) to 15 (≥13 defined as HG), and the questionnaire also includes one Quality of Life (QOL) question which is scored from 0 (poor) to 10. A Norwegian study describes high PUQE-scores (median 13) and low QOL score (median 3) when HG patients were admitted to hospital [23]. Following treatment (antiemetics and fluid-/nutritional regimens) the PUQE-score decreased and QOL score increased to the same levels as those found in a control group of healthy pregnant women. Thus hospital treatment reduces the burden of disease on an individual level.

### How does this disease burden relate to economic costs?

A Canadian study [24] estimated direct (medication, hospital/health care cost) and indirect costs (sick leave) of 139 women calling their NVP helpline. For women classified as severe NVP this amounted to $653 (Canadian dollar) total weekly cost.

They calculated a mean 23 days lost from work per woman. This is significantly more than the sum of hospital days and post-hospital rest days noted in this present study from Konikoff and coworkers (6 days) [10]. Since the Konikoff study did not take into account any sick-leave prior to the hospital stay or after the advised rest-days, we suspect that the actual cost is even larger than the estimate from the Konikoff-study.

Piwko also performed a US estimate of direct and indirect cost from HG [25]. This totaled as $ 185 millions (USD) or $7,089 per woman in 2012.

An estimate of annual hospital costs due to HG in England in 2003/2004, based on National Health Service reports on mean hospital stay of 3.5 days per admission, totalled £ 36.5 million (GBP, approximately 53.3 USD) [26]. In that same review, the authors described an almost three-fold rise in hospital admissions due to HG from 1989/99 until 2005/2006, explained mainly by the lack of early treatment of NVP in England in those years. Thus the Israeli report from Konikoff [10] are in line with those from Canada, England, Israel and USA in underscoring HG as having substantial economic implications for society.

### How can the burden of HG possibly be reduced?

- Decrease incidence by initiating proper antiemetic treatment.

In general, HG is understood as the extreme variant of NVP [26]. Thus, treating symptoms of NVP at an early stage may prevent development of the severe form. No large randomized controlled study has been performed evaluating whether early initiation of antiemetic treatment may reduce the incidence of HG. However, ecologic studies indirectly indicate this, due to the fact that when a former widely used medication approved for treatment of NVP was retracted from the US marked in 1983, the hospitalization due to HG steeply increased [27].

Hyperemesis in a former pregnancy is the strongest risk factor for developing HG; primigravidas have a 1 % incidence of HG while women who suffered from HG in a former pregnancy have 15 % recurrence risk (Odds Ratio 26) as opposed to women without HG in their first pregnancy have a 0.7 % risk of developing HG in a following pregnancy [28]. One randomized controlled study where 60 women who suffered from severe NVP in a former pregnancy were allocated to either start on antiemetics immediately when their pregnancy was recognized or start when symptoms of NVP developed. In the preemtive group 15 % developed severe NVP (PUQE ≥ 11) while in the symptom-based treatment group 39 % developed severe NVP [29].

We propose that antiemetic treatment could contribute to the reduction of expenditures due to HG by reducing the incidence as well as severity of HG.

- Day care treatment rather than hospital admission is feasible and less costly.

In general women with HG will be admitted to hospital for rehydration and nutritional therapy.

Two randomized studies allocating women with moderate/severe NVP to rehydration as inpatient or outpatient both demonstrated significantly shorter length of hospital stay of the day-care group but with similar satisfaction scores [30, 31]. Using the latter study in a Markov model for cost utility analyses, Murphy and collaborators found day-care treatment as significantly less costly; €985 (EUR approximately 1,205 USD) versus €3837 (approximately 4,692 USD) for in-patient treatment [32].

- Nutritional treatment by tube feeding is less costly than parenteral nutrition and may be continued as out-patient

Although first line treatment of HG patients is based on antiemetics and rehydration, these patients are at risk of severe under-nutrition. A South-African and a Norwegian study both document very low 24-h nutritional intake in HG patients (median 1035 kcal and 990 kcal respectively) [23, 33]. Parenteral nutrition (by venous catheters) carries risks of serious complications such as sepsis, thrombosis and pneumothorax and will usually need hospitalization [34, 35]. In a 10-year hospital cohort Stokke and collaborators [36] identified 107 patients (out of 558 women hospitalized due to HG) who were treated with tube feeding and documented their reversal of weight loss. 58 of these women continued tube feeding at home after discharge. In general, enteral nutrition (tube feeding) is less costly than parenteral nutrition [37], but cost-benefit analyses regarding these treatment modalities in HG are lacking.

### Directions for further research

Several factors hamper HG research:

- We need a consensus on the definition of HG and on core outcomes in order to be able to compare different studies.
- Larger placebo-controlled studies are needed to determine efficacy and side effects of treatments. At present there is insufficient evidence to prefer any specific antiemetic regimen.
- Antiemetics should be evaluated specifically for NVP/HG, with a focus on the risk of malformations and other possible fetal effects are important. Even though there is substantial evidence of no increased risk from antihistamines, these are still not formally approved as treatment of NVP or HG in several countries.
- The role of nutritional support for HG patients should be clarified.
- All effects of treatment should be evaluated on an individual level (patient satisfaction), as well as in relation to the economic burden for society.
- Long as well as short term maternal and fetal complications due to nutritional deficiencies should be explored.

## Conclusion

Hyperemesis gravidarum is a condition with substantial economic burden in addition to severe physical and psychological suffering. This disease needs to be further explored, and we suggest a major focus on modifiable risk factors and better treatment regimen. With improved therapy hopefully we can reduce the burden for affected women as well as society.

## Abbreviations

EUR: Euro
GBP: British pound
HG: Hyperemesis gravidarum
NVP: Nausea and vomiting of pregnancy
PUQE: Pregnancy unique questionnaire of emesis
US: United States of America
USD: American dollars
